# Recurrent Acute Methemoglobinemia in an Infant With Persistent Gastroenteritis: A Case Report

**DOI:** 10.7759/cureus.51909

**Published:** 2024-01-08

**Authors:** Nawarah A Alhamoud, Ryan A AlZugaibi, Waad M Aldoohan

**Affiliations:** 1 Medicine, College of Medicine, King Saud Bin Abdulaziz University for Health Sciences, Riyadh, SAU; 2 Pediatric Hematology Oncology, King Abdullah Specialized Children's Hospital, Riyadh, SAU; 3 Hematology, Ministry of National Guard Health Affairs, Riyadh, SAU

**Keywords:** cyanosis, well water, gastroenteritis, methemoglobin, methemoglobinemia

## Abstract

Acute toxic methemoglobinemia is a rare and fatal condition with increased levels of oxidized hemoglobin. The clinical presentation of methemoglobinemia varies primarily based on total methemoglobin levels in the blood. Patients sometimes have significant cardiopulmonary compromise, but the majority are asymptomatic, with only cyanosis as the most prevalent sign. We report the case of a 41-day-old male who developed methemoglobinemia and persistent gastroenteritis after consumption of well water. In this case, we believe that the recurrence of acute methemoglobinemia episodes resulted from multifactorial reasons such as age at presentation, infection with nitrate-producing organisms, and consumption of nitrite-containing well water. The rationale for prophylactic therapy was implemented, aiming to prevent further episodes. This case report demonstrates the potential of prophylactic therapy as part of the management of infants with recurrent acute methemoglobinemia episodes.

## Introduction

Methemoglobinemia is a rare disorder associated with a hypoxic state. Unlike hemoglobin, methemoglobin cannot bind oxygen owing to its oxidized ferric state, leading to what is known as functional anemia. The severity of its presentation depends on the total amount of methemoglobin in the blood, and cyanosis usually develops when total methemoglobin levels exceed 1.5 g/dL [1]. Methemoglobinemia manifests with a constellation of symptoms, including central nervous system (CNS) depression, seizures, coma, cyanosis, pallor, fatigue, metabolic acidosis, and death [1].

Methemoglobinemia can either be acquired or inherited. Inherited methemoglobinemia can result from cytochrome b5 reductase deficiency owing to autosomal recessive pathogenic variants in the cytochrome b5 reductase 3 (CYB5R3) gene and is considered the rarest form of the disease. The CYB5R3 gene serves as an electron donor in the process of converting methemoglobin to hemoglobin [2]. Hemoglobin M (Hb M) disease occurs because of autosomal dominant pathogenic variants affecting the alpha, beta, or rarely gamma globin genes. The pathophysiology behind this is a consequence of substituting tyrosine for histidine, which leads to the formation of an iron-phenolate complex. This complex prevents the reduction of ferric iron to its ferrous state, resulting in chronic methemoglobinemia. Individuals who have Hb M disease are asymptomatic aside from cyanosis [3]. The acquired form of methemoglobinemia is the most common, being a result of exposure to exogenous substances or toxins that oxidize hemoglobin [2]. Such exogenous toxins include those from nitrite-forming bacteria such as *Escherichia coli* (*E. coli*) and *Campylobacter*. They induce methemoglobinemia by providing a high nitrite load that, in turn, oxidizes hemoglobin’s ferrous iron to a ferric state, leading to the formation of methemoglobin [3, 4]. Another example is well water, which is hazardous because of its high nitrate content (100 ppm) that oxidizes hemoglobin and results in methemoglobinemia [5].

## Case presentation

Our patient was a 41-day-old male, full-term, with an unremarkable perinatal and postnatal history. He presented to the emergency department (ED) with fever, diarrhea, and vomiting for three days. He was admitted as a case of acute gastroenteritis. The polymerase chain reaction (PCR) of the nasopharyngeal swab was positive for human adenovirus and rhino/enterovirus. During admission, the patient had an electrolyte imbalance and hyperchloremic metabolic acidosis, which improved with hydration. Subsequently, he showed some improvement and was discharged after a few days. After one day of discharge, he presented again to the ED with persistent diarrhea and hypoactivity. He had cyanosed lips and oxygen desaturation. Supportive management with oxygen, fluids, and antibiotics was administered. Chest X-rays and echocardiography were normal and did not show the cause of the cyanosis episode. Venous blood gas (VBG) showed metabolic acidosis and a high methemoglobin level of 21%. The patient’s clinical condition, oxygen requirement, and cyanosis significantly improved after one dose of methylene blue, and methemoglobin levels returned to the normal range. After further history-taking, the mother mentioned that well water was used in formula preparation. The patient had no family history of similar presentations or congenital cyanosis. Gastrointestinal multiplex PCR from stool revealed two pathogens: *Campylobacter* species and *E. coli*. After completing a course with third-generation cephalosporins, ceftazidime, and azithromycin, his hemolytic workup, including hemoglobin electrophoresis, reticulocyte count, total bilirubin, lactate dehydrogenase, and glucose-6-phosphate dehydrogenase (G6PD) levels, were within the normal range for his age. The patient showed improvement with time and was discharged after a few days in a stable condition, with advice to avoid well water consumption.

Three weeks after the second admission, he presented to the ED. He again had central cyanosis and oxygen desaturation. The VBG test revealed metabolic acidosis with a high methemoglobin level of 24.9%. He responded well to the methylene blue administration and was admitted for observation. The patient still experienced profuse diarrhea. Repeated gastroenteritis multiplex PCR showed shedding of the same organisms seen in the previous admission (*Campylobacter *species and* E. coli*). After four days of admission, he developed another episode of cyanosis and desaturation, which again responded well to methylene blue (Table 1).

**Table 1:** Pre- and post-methylene blue administration venous blood gas (VBG) clinical and laboratory results O_2_: oxygen; Met Hgb: methaemoglobin; pH: potential hydrogen; HCO_3_: bicarbonate

	Pre-methylene blue				Post-methylene blue	
	Oxygen saturation	Met Hgb (total)	pH	HCO_3_	Oxygen saturation	Met Hgb
First episode	90%, on 5 L/m O_2_	21.0% (1.97 g/dL)	7.329	14.5 mmol/L	98%, on room air	2.6%
Second episode	93%, on 2 L/m O_2_	24.9% (1.97 g/dL)	7.285	12.6 mmol/L	100%, on room air	3.3%
Third episode	86%, on 10 L/m O_2_	16.7% (1.65 g/dL)	7.274	7.3 mmol/L	100%, on room air	2.4%

Given the continuous profuse diarrhea, the patient was treated with ciprofloxacin due to possible azithromycin resistance. Riboflavin and ascorbic acid were administered as prophylactic therapy to prevent further acute methemoglobinemia episodes. Continued care was implemented for two weeks with hydration and correction of acidosis, and the patient showed significant improvement in the diarrhea. The immunological workup was negative for possible immunodeficiency. He received a transfusion of packed red blood cells twice during admission.

Following up at the pediatric hematology clinic a month later, the patient was doing well clinically, with normal stool consistency and methemoglobin levels. Whole-exome sequencing (WES) was negative for common causes of congenital methemoglobinemia. Given that congenital causes of methemoglobinemia were unlikely and secondary causes were controlled, ascorbic acid and riboflavin administration with a tapering schedule was continued for three months. Ascorbic acid was stopped after two months of therapy when a renal ultrasound showed increased bilateral pyramidal echogenicity, suggesting early medullary nephrocalcinosis. Riboflavin was stopped three months after the last episode, as planned. Currently, four months have passed since the last episode, and the patient is doing well with no cyanosis or acute toxic methemoglobinemia episodes.

## Discussion

Methemoglobinemia is a rare, life-threatening condition that presents with cyanosis and requires prompt attention. The development of cyanosis is correlated with the total amount of methemoglobin rather than its percentage in the blood [1]. Infancy is critical in terms of developing methemoglobinemia because of the predominance of fetal hemoglobin, which is easily affected by oxidative stress and, consequently, increases the susceptibility to methemoglobinemia [5]. Moreover, infants under three months of age have an erythrocyte cytochrome b5 reductase activity of less than 50%. This decrease in the function of the enzymatic systems responsible for the reduction of methemoglobin results in methemoglobinemia [6]. Another factor that makes infants more susceptible to developing methemoglobinemia is their higher intestinal pH. This may promote the proliferation of intestinal flora, which, in turn, potentiates the conversion of nitrates to nitrites from dietary intake. Nitrate poisoning poses a significant risk to infants who consume water from a well [5]. Our case is consistent with those described in the literature, which emphasizes the harmful effects of well water consumption during infancy and highlights a potential source for this condition [5]. Well water may also be a source of infection as it provides optimal conditions for bacterial growth. Greer et al. reported that gastroenteritis is a predisposing factor to methemoglobinemia, especially in infants younger than three months of age. Indeed, the authors suggested an association between methemoglobinemia and enteritis, as 49% of their patients had persistent diarrhea [6].

Our patient had persistent gastroenteritis secondary to *E. coli* and *Campylobacter *infections. We believe the reasons for the recurrent episodes of acute methemoglobinemia are multifactorial: (1) being an infant less than three months of age; (2) consumption of formula prepared with well water; and (3) having persistent and profuse diarrhea due to an infection by gut pathogens that contribute to the development of methemoglobinemia by converting nitrates into nitrites in the gut [6]. Therefore, treatment of the underlying cause with antibiotics probably played a major role in the prevention of subsequent episodes of acute toxic methemoglobinemia. The management of methemoglobinemia in a child or infant is dependent on numerous factors, such as age, total methemoglobin, and whether or not the patient is symptomatic [6].

Methylene blue is the preferred choice in the treatment of acute toxic methemoglobinemia. It is indicated when methemoglobin levels are greater than 30% and for symptomatic patients with methemoglobin levels ranging between 20% and 30%, particularly those with signs of cardiac or pulmonary comorbidities [1]. The cosmetic use of methylene blue is also indicated to alleviate cyanosis [7]. In addition to methylene blue, preventive measures such as the administration of riboflavin and ascorbic acid are also used in acute cases for cosmetic purposes [7].

Ascorbic acid (vitamin C) improves cyanosis by acting as a reducing agent, leading to the reduction of methemoglobin to hemoglobin [1, 8]. Its use is beneficial in cases of methemoglobinemia when methylene blue is contraindicated, such as in G6PD, or when methylene blue is unavailable [8]. Furthermore, riboflavin (vitamin B2), via the nicotinamide adenine dinucleotide-flavin reductase system, accelerates the reduction of methemoglobin levels by acting as an electron donor, thereby reducing methemoglobin [1, 9]. In a case series, ascorbic acid was administered to five patients with methemoglobinemia, resulting in the resolution of cyanosis and full recovery after 24 hours of treatment [8].

In this case report, we describe our approach to managing an infant with recurrent methemoglobinemia episodes and our prophylactic therapy plan to prevent recurrence.

## Conclusions

Episodes of acute toxic methemoglobinemia are lethal if left untreated, and supportive measures, along with methylene blue, should be implemented rapidly. Methemoglobinemia can either be inherited or acquired. In acquired cases of methemoglobinemia, control of the underlying cause is the mainstay of treatment. In the case of recurrent episodes of acute methemoglobinemia in high-risk patients, prophylactic therapy with ascorbic acid and/or riboflavin might be considered; however, monitoring the medications’ side effects and offering a clear tapering plan is necessary to avoid complications. Studies on a larger scale are needed to support the use of prophylactic measurements safely and effectively.
